# Identification of Light-Independent Anthocyanin Biosynthesis Mutants Induced by Ethyl Methane Sulfonate in Turnip “Tsuda” (*Brassica rapa*)

**DOI:** 10.3390/ijms18071288

**Published:** 2017-06-22

**Authors:** Jian-Fei Yang, Yun-Zhu Chen, Saneyuki Kawabata, Yu-Hua Li, Yu Wang

**Affiliations:** 1 College of Life Science, Northeast Forestry University, Harbin 150040, China; yjf19910304@126.com (J.-F.Y.); chenyunzhu_csuft@163.com (Y.-Z.C.); 2 Graduate School of Agricultural and Life Sciences, The University of Tokyo, Yayoi, Bunkyo Tokyo 113-8654, Japan; ayuki@mail.ecc.u-tokyo.ac.jp; 3 State Key Laboratory of Tree Genetics and Breeding, Northeast Forestry University, Harbin 150040, China

**Keywords:** anthocyanin, EMS mutant, turnips, light

## Abstract

The epidermis of swollen storage roots in purple cultivars of turnip “Tsuda” (*Brassica rapa*) accumulates anthocyanin in a light-dependent manner, especially in response to UV-A light, of which the mechanism is unclear. In this study, we mutagenized 15,000 seeds by 0.5% (*v*/*v*) ethyl methane sulfonate (EMS) and obtained 14 mutants with abnormal anthocyanin production in their epidermis of swollen storage roots. These mutants were classified into two groups: the *red* mutants with constitutive anthocyanin accumulation in their epidermis of storage roots even in underground parts in darkness and the *white* mutants without anthocyanin accumulation in the epidermis of storage roots in aboveground parts exposed to sunlight. Test cross analysis demonstrated that *w9*, *w68*, *w204*, *r15*, *r21*, *r30* and *r57* contained different mutations responsible for their phenotypic variations. Further genetic analysis of four target mutants (*w9*, *w68*, *w204* and *r15*) indicated that each of them was controlled by a different recessive gene. Intriguingly, the expression profiles of anthocyanin biosynthesis genes, including structural and regulatory genes, coincided with their anthocyanin levels in the epidermis of storage roots in the four target mutants. We proposed that potential genes responsible for the mutations should be upstream factors of the anthocyanin biosynthesis pathway in turnips, which provided resources to further investigate the mechanisms of light-induced anthocyanin accumulation.

## 1. Introduction

Anthocyanins, synthesized through the flavonoid pathway, are important secondary metabolites for plants flower pigmentation, seed dispersal, fruits coloration and resistance against biotic or abiotic stresses [1,2,3,4]. Due to high antioxidant activity, anthocyanins are critical antioxidants to protect plants against accumulation of reactive oxygen species (ROS), as observed in rats and humans [5,6]. Bright organ colors derived from anthocyanin accumulation directly determine ornamental, diet and market values of fruits and ornamental crops. Therefore, a comprehensive understanding of mechanisms regulating anthocyanin biosynthesis is important for the bioengineering of anthocyanin production in ornamental and agricultural plants.

The biosynthetic pathways of anthocyanin in different species share a majority of common routes that have been well characterized [7,8,9]. The corresponding genes have been isolated from various anthocyanin mutants and natural variant plants [10,11,12,13]. In the biosynthetic pathway of anthocyanin, chalcone synthase (*CHS*) is one of the first determinants of anthocyanin biosynthesis, which collaborates with other structural genes, such as chalcone isomerase (*CHI*), flavanone 3-hydroxylase (*F3H*), dihydroflavonol reductase (*DFR*), anthocyanidin synthase (*ANS*) and UDP-flavonoid glucosyl transferase (*UFGT*), to synthesize anthocyanins [14,15,16]. These genes are divided into two subgroups: early biosynthetic genes (EBGs) for production of flavonols and late biosynthetic genes (LBGs) leading to the production of anthocyanins in vegetative tissues. In *Arabidopsis*, activation of EBGs requires co-activator independent R2R3-MYB regulatory genes (*MYB11*, *MYB12* and *MYB111*), whereas LBGs are activated by R2R3-MYB genes and basic helix-loop-helix (bHLH) proteins that form a MYB-bHLH-WD40 (MBW) transcriptional activation complex with a WD40 repeat protein [17,18]. The members of this regulatory complex consist of TRANSPARENT TESTA GLABRA 1 (TTG1); the R2R3-MYB proteins from PRODUCTION OF ANTHOCYANIN PIGMENT 1 (PAP1/MYB75), PAP2, MYB113, or MYB114; and the bHLH proteins from TRANSPARENT TESTA 8 (TT8), GLABRA3 (GL3), or ENHANCER OF GLABRA3 (EGL3). The formation of MBW complex has been demonstrated to play a key role in triggering anthocyanin accumulation [19].

The anthocyanin biosynthesis is affected by many different environmental factors, of which light is a critical one [20,21,22]. Light-regulated anthocyanin biosynthesis initiates by a group of plant photoreceptors perceiving light signals of different wavelengths and then transducing signals to promote expression of anthocyanin biosynthesis genes via intracellular second messenger systems. The signal transduction pathway of light-dependent anthocyanin biosynthesis involves at least three types of photoreceptors: the red and far-red light-sensing phytochromes, the blue/ultraviolet A perceiving cryptochromes, and the UV-B specific photoreceptor UV RESISTANCE LOCUS8 (UVR8) [23,24,25,26,27]. As a positive regulator of photomorphogenesis, ELONGATED HYPOCOTYL 5 (HY5) is required for light-induced anthocyanin accumulation in plants [28]. Although there is no physical interaction between HY5 and R2R3-MYB proteins (such as MYB12, MYB111 and PAP1), the expression level of these MYB factors is activated by light in a HY5-dependent manner [29,30]. CONSTITUTIVELY PHOTOMORPHOGENIC 1 (COP1) is another main player in the integration of photomorphogenesis and anthocyanin biosynthesis. Especially, most of regulatory factors involved in anthocyanin production (such as PAP1 and PAP2) are substrates of COP1/SPA, which are E3 ubiquitin ligases. The *cop1* and *spa* mutants, but not *PAP1* over-expressing plants, are able to produce anthocyanins in dark-grown seedlings, indicating that light requirement for anthocyanin biosynthesis is a result of the light-mediated stabilization of PAP1 proteins [31]. However, besides HY5-COP1/SPA-R2R3MYB module, more knowledge about other regulators involved in anthocyanin biosynthesis in response to light needs further investigation.

Turnips (*Brassica rapa*) are economically important vegetable crops in the world with enriched nutrition and medicinal value in their swollen roots. Previous work has indicated that UV-A robustly induced anthocyanin accumulation in the epidermis of swollen storage root, on which single blue light showed no effect [32]. The mRNA levels of *CHS* remarkably increased after 6 h of UV-A treatment compared to other monochromatic lights, such as red, blue and UV-B, which is distinguished from the cryptochromes and UVR8 reactions in *Arabidopsis*. In addition, UV-A has been proved to exert different effects on induction of anthocyanin accumulation in hypocotyls of turnip seedlings [33]. In *Arabidopsis*, cryptochromes are considered as UV-A/blue light photoreceptors that play critical roles in regulating blue light-mediated photomorphogenic growth and developmental process. However, different responses to blue light and UV-A irradiation for anthocyanin accumulation in turnip “Tsuda” showed probability of distinct mechanisms, besides cryptochrome signal transduction pathway, regulating anthocyanin biosynthesis in response to UV-A [32].

In the last decade, mutagenesis was used to obtain a large number of mutants that accelerated study of many different signal transduction networks. Ethyl methane sulfonate (EMS) has been the most common mutagen of choice to establish a series of allelic mutations in all genes with desired identifiable characters in plants such as plant height, flowering time, leaf shapes and fruit color changes [34,35,36,37]. It produces random mutations in genetic material by nucleotide substitutions that primarily cause point mutations through alkylation on the O^6^ position of guanines. Concentration of EMS is the most important factor that determines mutagenesis efficiency. In general, a EMS concentration yielding 50% seed lethality (LD_50_) is defined as an indicator for population development to obtain a large number of desirable mutations in many plants [37,38,39].

To identify novel genes associated with light-induced anthocyanin accumulation in turnip “Tsuda”, we mutagenized 15,000 wild-type (WT) seeds using an optimal concentration of EMS and observed several different phenotypes in M_2_ plants. Totally, 14 mutants with phenotypic changes of anthocyanin production in the epidermis of swollen storage roots were obtained after extensive screening. The test cross and backcross experiments in genetic analysis indicate that four light-independent anthocyanin mutants (*w9*, *w68*, *w204* and *r15*) are caused by a different recessive gene. qRT-PCR analysis showed that anthocyanin biosynthesis genes were dramatically up-regulated in *r15* and down-regulated in *w9, w68* and *w204,* compared to these of in WT plants. In this study, we obtained a serial of stably inherited anthocyanin mutants for researches on light-induced anthocyanin accumulation, which may provide new insight into the network of anthocyanin biosynthesis in higher plants.

## 2. Results

### 2.1. Characterization of Turnip “Tsuda” (Brassica rapa)

**Figure 1:**
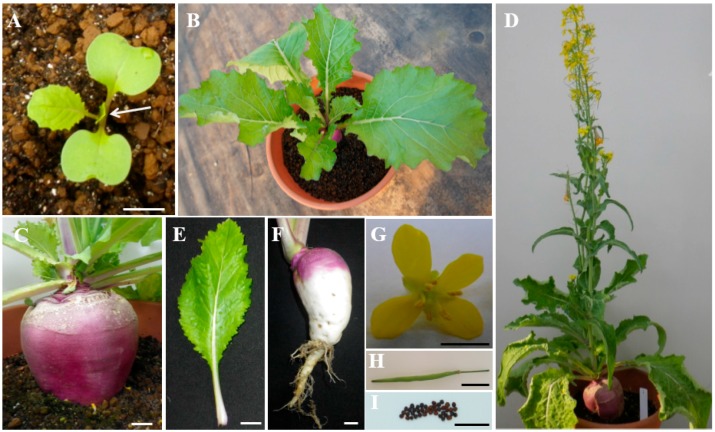
Turnip cultivar “Tsuda” plant development: (**A**) turnip seedlings; (**B**) vegetative growth of plants; (**C**) root swelling stage; (**D**) main shoot of flowering plant; (**E**) fully expanded rosette leaf; (**F**) light-induced anthocyanin accumulation in swollen root; (**G**) bright-yellow flower; (**H**) silique; and (**I**) mature seeds. The white arrow in (**A**) indicates the growing point of seedlings. Bar = 0.5 cm.

Turnip belongs to species of *Brassica* and subspecies of *Brassica rapa* in *Brassicaceae* family. Purple turnip “Tsuda” is a biennial herb and mature plants need to be vernalized for blooming in winter. Vegetative stage of turnip begins approximately 5–10 days after sowing, while cotyledons of seedlings emerge above soil surface and expand to initiate leaf growth from the growing point (Figure 1A). With the sixth leaf growing, the root of turnip initiates swelling up to 4–5 cm in diameter (Figure 1B,C). During winter, low temperature contributes to vernalization of turnip and transforms turnip from vegetative stage to productive stage (Figure 1D). Besides swollen storage root and seed coat, other tissues of turnip are similar to *B.rapa* oilseed crops and barely accumulate anthocyanins (Figure 1E–I). Furthermore, the epidermis of swollen storage roots of turnip cultivar “Tsuda” accumulate anthocyanins in a light-dependent manner, which only pigment at the light exposed part rather than that in darkness covered with soil. Therefore, it is an attractive system for fundamental research of mechanism of light-induced anthocyanin biosynthesis in plants.

### 2.2. Optimization of Mutagen Dosage

Optimal mutagenesis will lead to an EMS mutant population with a high density of mutations but also vigorousness and fertility. To determine the optimal concentration of EMS for turnip mutagenesis, we used 200 seeds for each of six treatments (0%, 0.25%, 0.50%, 1%, 2%, and 5%) and calculated germination rates (GR) of treated seeds (Table 1). The results showed that the GR of seeds decreased with increasing EMS concentrations. Compared to 191 seeds of the control group (no EMS), the GR of seeds were significantly reduced to less than 20% under 1.0% EMS treatment and down to 0% with EMS concentration over 2%. These findings indicated that the EMS treatment had been effective on turnip seeds and higher EMS concentrations increased the probability of undesirable mutations with more lethality. Almost 51% seeds were germinated by 0.5% EMS treatment, the closest to LD_50_ among all treatment conditions. Thus, we used 0.5% EMS to induce mutagenesis in over 15,000 turnip seeds.

### 2.3. Screening Light-Independent Anthocyanin Mutants Induced by EMS

**Figure 2:**
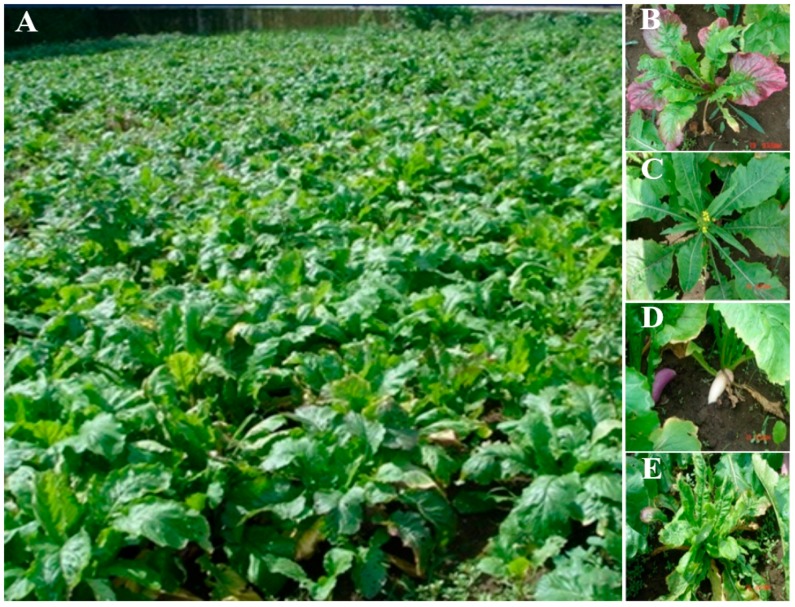
Open field cultivation and phenotypic variations observed in M_1_ population: (**A**) open field cultivation of M_1_ population generated by EMS; and phenotypic characteristics of abnormal turnips with: purple leaf (**B**); early flowering (**C**); white swollen root (**D**); and curly leaf (**E**).

We planted EMS-treated seeds in field to survey phenotypic variations in M_1_ generation (Figure 2A), and observed many types of abnormal phenotypes: purple color leaves, early flowering, white swollen root peels and curled leaves in different developmental stages of M_1_ population (Figure 2B–E). Among all treated seeds, 56.7% (8516) M_1_ plants were generated to reach maturity, 3.4% (291) of which did not germinate or produced viable plants in M_2_ population. Totally, we obtained 14 putative mutants that displayed phenotypic variations in anthocyanin accumulation in the epidermis of swollen storage roots from the M_2_ population. Among these mutants, eight plants (*r1*, *r10*, *r15*, *r21*, *r30*, *r37*, *r53* and *r57*) constitutively accumulated anthocyanin even in the epidermis of swollen storage roots at underground parts (with no light installed) and were designated as *red* mutants; and six plants (*w1*, *w3*, *w9*, *w68*, *w146* and *w204*) displayed deficient anthocyanin production in the epidermis of swollen storage roots at both aboveground (exposed to sunlight) and underground parts and were designated as *white* mutants.

To confirm whether these mutants were affected by same genes to display phenotypic variations, we performed test cross experiments between M_3_ generation of mutants on same phenotypes and recorded phenotypes of their progenies. The results of F_1_ progeny generated by six *white* mutants showed that *w68*, *w204* and *w9* were caused by different mutated loci (Table S1). However, three progenies of the crossed *red* mutants showed mutation phenotypes as their parental strains (Table S2). The results indicated that *r10*, *r15* and *r57* probably attributed to mutations of the same gene induced by EMS. Additionally, the phenotypic variations of *r15*, *r21*, *r30* and *r37* were caused by mutations of different genes.

**Figure 3:**
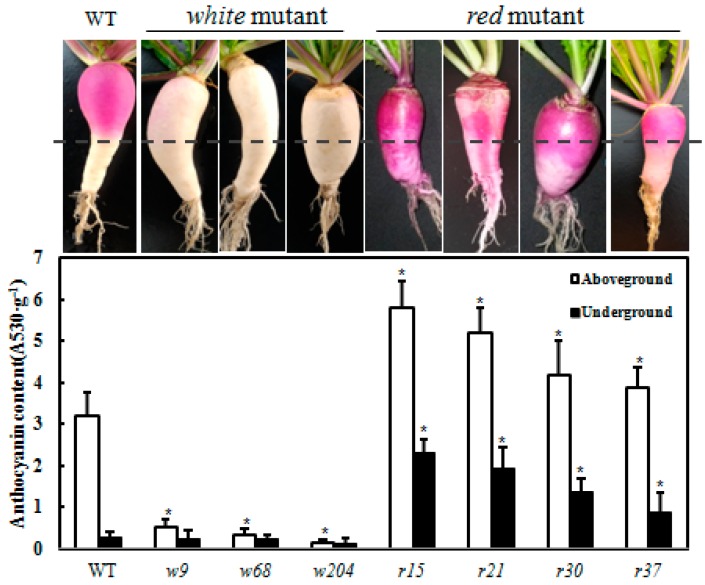
Characterization of light-independent anthocyanin mutants. Phenotypes of the WT and mutants isolated in M_2_ population are shown. *White* mutants with anthocyanin deficiency (*w9*, *w68* and *w204*); *Red* mutants with anthocyanin over accumulation in the epidermis of swollen storage roots (*r15*, *r21*, *r30* and *r37*). The swollen roots under dash line were covered by soil, while the parts above dash line were exposed to sunlight. Total anthocyanin content in mutants and WT were measured. The white columns, aboveground part of epidermis of swollen roots; black columns, underground part of epidermis of swollen roots. The data represent mean values from three replicates with biological repeats (*n* ≥ 3). Error bars represent the standard error of the mean. “*” represents significantly differences at the level of *p* < 0.01.

To examine the molecular basis of light-independent anthocyanin accumulation in these EMS mutants, we measured the anthocyanin content of each mutant. In WT plants, pigmentation in the epidermis of swollen storage roots showed a light inducible manner: anthocyanin accumulated only in the aboveground part of storage roots (Figure 3). In *white* mutants *w9*, *w68* and *w204*, anthocyanin was undetectable in either the aboveground or underground parts of storage roots. In contrast to *white* mutants, the *red* mutants (*r15*, *r21*, *r30* and *r37*) accumulated anthocyanin in both underground and aboveground parts of storage roots (Figure 3). However, we noticed that anthocyanin levels of underground parts were lower than aboveground parts in *red* mutants as well as that in WT. These data suggest that anthocyanin biosynthesis of these *red* mutants is partially regulated by light.

### 2.4. Genetic Analysis of the Anthocyanin Variations Mutants

**Figure 4:**
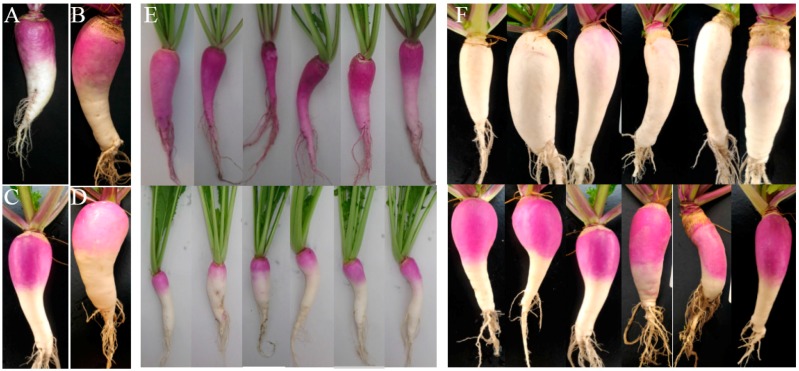
Phenotypes of F_1_ and F_2_ populations in different mutant lines. F_1_ generation of: *r15* (**A**); *w9* (**B**); *w68* (**C**); and *w204* (**D**). Ten individuals of each line were collected for observation. Partly F_2_ individuals of: *r15* (**E**); and *w68* (**F**).

We constructed F_2_ populations by crossing the M_3_ generation of each mutant line (*r15*, *w9*, *w68* and *w204*) with WT. The derived F_1_ generation of four mutants showed similar phenotype to WT (Figure 4A–D). The F_1_ plants were self-fertilized to produce F_2_ progeny. In the F_2_ population, including over 150 individuals, two phenotypes segregated in the ratio of 3:1 (Figure 4E,F). Furthermore, the *p* value of χ^2^ test in each of four groups was more than 0.05, which fits the 3:1 ratio expected for one-locus segregation (Table 2). These results indicated that different single recessive genes controlled the mutation traits in *w9*, *w68*, *w204* and *r15* mutants.

### 2.5. Scanning for Mutations by TILLING Analysis

**Figure 5:**
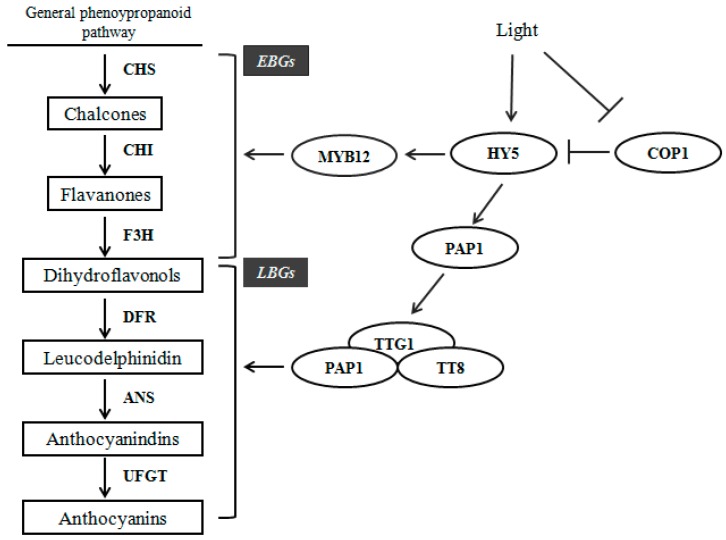
Anthocyanin biosynthesis pathways in turnips “Tsuda”. Enzymes encoded by flavonoid early structural genes (EBGs) and encoded by anthocyanin late structural genes (LBGs) are shown in bold. Products generated by each step are boxed. Regulatory genes are circled. These genes control structural genes expression to accumulate anthocyanins in a HY5-dependent manner under light.

**Figure 6:**
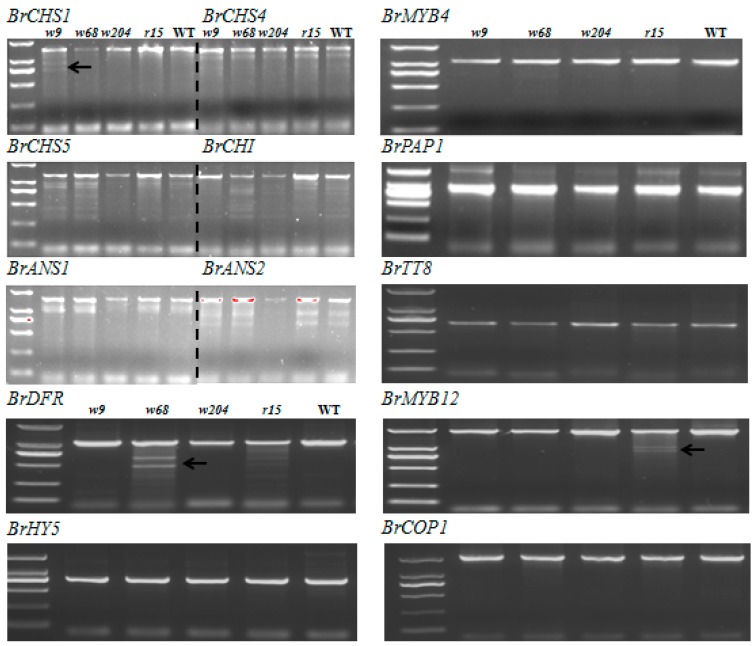
TILLING analysis of anthocyanin biosynthesis genes and light responsive genes in *white* mutants and *red* mutants. Each sample was mixed by equal amounts of mutants and WT PCR products via specific primers amplification and digested by *Cel*I, respectively. Seven mutants (*w9*, *w68*, *w204*, *r15*, *r21*, *r30* and *r37*) of treated samples were loaded from left to right in 1.5% agarose gel for mutation scanning. Black arrows indicate *Cel*I-digested fragments.

TILLING (Targeting Induced Local Lesions in Genomes) is a widely used approach to determine target genes associated with specific mutation in mutant populations generated by EMS treatment. In turnip, structural genes *BrCHS1*, *BrCHS4*, *BrCHS5*, *BrCHI*, *BrF3H*, *BrANS*, *BrDFR* and *BrUFGT* promote anthocyanin biosynthesis in response to light. The regulatory factors *BrMYB12*, *BrPAP1*, *BrTT8*, and *BrTTG1* have positive effects on their target structural genes to regulate anthocyanin production, of which expression levels are partially influenced by light in a BrHY5-dependent manner (Figure 5). Therefore, sequences of anthocyanin biosynthesis genes (including structural genes and regulatory genes) and light responsive genes *BrHY5* and *BrCOP1* from mutants and WT plants were amplified for point mutation screening. From TILLING analysis, we detected a nonsense mutation occurred in the second exon of *BrMYB4* in *r30* [40]. Several mutations located in other anthocyanin synthesis genes were also founded. However, all of them were identified as synonymous mutations, such as *BrCHS1* in *w9*, *BrDFR* in *w68* and *BrMYB12* in *r15* (Figure 6). Taken together, these results suggested that the mutant phenotypes in these lines are likely due to mutations of factors other than the anthocyanin biosynthetic genes.

### 2.6. Expression Profiles of Anthocyanin Biosynthetic Genes

**Figure 7:**
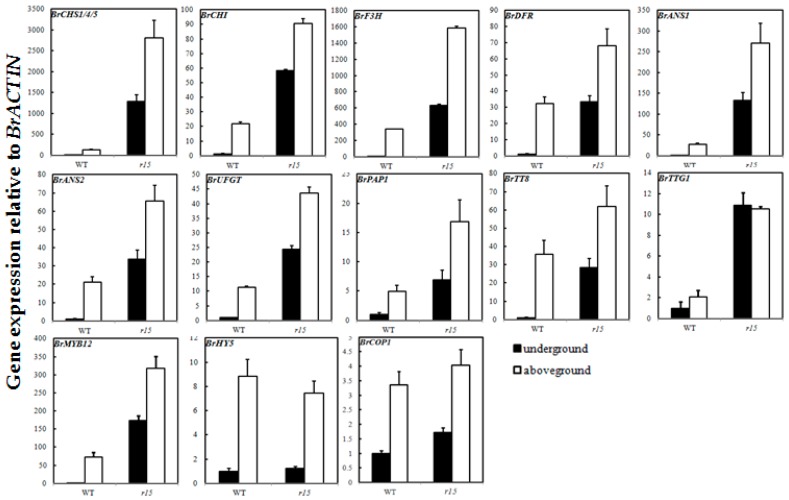
Expression analysis of anthocyanin biosynthesis genes and light responsive genes in underground and light-exposed parts of epidermis of swollen roots in *r15* and WT. The expression value for each sample was normalized to that of the *BrACTIN* gene, and the transcript level in underground parts of epidermis of swollen roots was set as 1.0. The white columns, aboveground part of epidermis of swollen roots; black columns, underground part of epidermis of swollen roots. The mean values of three biological replicates are shown with error bars (SD).

**Figure 8:**
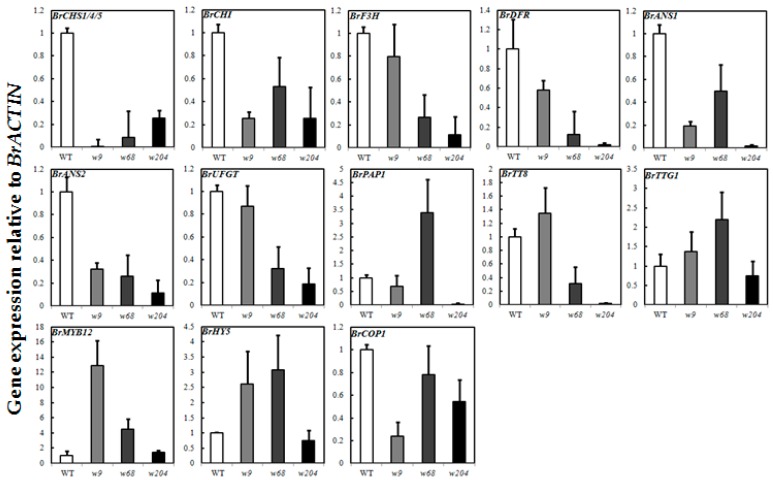
Expression analysis of anthocyanin biosynthesis genes and light responsive genes in light-exposed swollen root peels of *white* mutants and WT. The expression value for each sample was normalized to that of the *BrACTIN* gene, and the transcript level in light-exposed epidermis of swollen roots of WT was set as 1.0. The expression levels of *w9* (gray column), *w68* (dark gray column) and *w204* (black column) were compared to WT (white column). The mean values of three technical replicates are shown with error bars (SD).

To further analyze the types of target mutants, we detected the expression levels of anthocyanin biosynthesis genes and light responsive genes of *B. rapa* in the epidermis of swollen storage roots (Table S3). In WT, anthocyanin biosynthesis genes displayed a light-dependent manner that increased 20–50 times in aboveground parts of swollen roots compared with those in soil (Figure 7). Transcript levels of all the structural and regulatory genes were much higher for *r15* than for the WT in the soil. However, in *r15*, expression levels of detected genes in aboveground parts of root were higher than those in underground parts, which also showed a light-dependent manner for anthocyanin accumulation like WT. *BrHY5* and *BrCOP1* are the upstream components of light-signaling pathway, of which the expression levels were similar between *r15* and WT. Except for *BrF3H* and *BrUFGT* in *w9*, expression of all target structural genes in *w9*, *w68* and *w204* was much lower than that in WT, corresponding to that of anthocyanin deficiency at aboveground storage roots. For the regulatory factors involved in anthocyanin biosynthesis, transcript levels of both *BrPAP1* and *BrTT8* were down-regulated in *w204*, while *BrPAP1* and *BrTTG1* expression was up-regulated and *BrTT8* was down-regulated in *w68*. The positive regulator *BrMYB12* and light responsive gene *BrHY5* exhibited higher transcript levels in *w9* and *w68*. Compared to WT, no change of light responsive genes *BrHY5* and *BrMYB12* expression was observed in *w204*, different from those in *w9* and *w68* (Figure 8). The expression level of *BrCOP1* in *w9* and *w204* were lower than that in WT, but showed no difference in *w68*. The systemic expression changes of anthocyanin related gene in these mutants further indicated that the mutated genes of *r15*, *w9*, *w68* and *w204* were likely to affect upstream components of anthocyanin biosynthesis to regulate light-induced anthocyanin accumulation.

## 3. Discussion

Previous studies have showed that anthocyanins in the epidermis of swollen storage roots of turnip “Tsuda” accumulated specifically in response to UV-A (320–400 nm, 3.0 Wm^−2^) and blue + UV-B (310 nm, 0.3 Wm^−2^), which is also observed in seedlings [32,33]. The expression of *BrCHS1*, *BrCHS4* and *BrCHS5* increased remarkably under both UV-A and blue + UV-B light, but not in response to single blue light (470 nm, 10 Wm^−2^). The transcriptome analysis indicated that there may be some overlaps between UV-A and blue + UV-B signal transduction pathways, in which 70% up-regulated genes were co-regulated, including some structural genes (*BrCHS*, *BrCHI*, *BrF3H* and *BrANS*) and regulatory genes (*BrPAP1*, *BrTT8* and *BrMYB12*) involved in anthocyanin biosynthesis pathway [41].

To gain additional insight into the mechanism of UV-A or blue + UV-B induced anthocyanin accumulation in turnip “Tsuda”, setting up a screen for mutants with light-independent anthocyanin accumulation is urgently needed. We employed a forward genetic screen approach because the genetic transformation is low efficiency with some malformed root-like structures developed when using tissue culture method. Hereby, we used EMS to generate a mutant population and screen for turnips with abnormal anthocyanin accumulation pattern in epidermis of swollen storage roots. For more effective mutagenesis, treatment with different EMS concentrations was carried out in a small scale. Several studies have been conducted to optimize EMS concentrations in constructing *Brassica* mutant libraries, with optimal EMS concentrations as 0.3–0.4% for *B. rapa* [42], 0.6% for *B. naups* [34,43,44] and 0.5% for *B. oleracea* [45]. These results indicate that 0.5% EMS is likely a suitable mutagenic concentration for *Brassica* species. Consistent with previous reports, our current study showed that 0.5% EMS were optimal for turnip “Tsuda” mutagenesis (Table 1). Meanwhile, rapidly reduction of germination was observed when the mutagen concentration increased, which may be due to absorbance of mutagen by the seeds leading to damage of germ cell or defects in cell metabolic processes.

Phenotypic changes in M_1_ generation are reliable signals for mutagen efficiency. Two types of visible changes on leaf and root peels coloration were related to anthocyanin production, which further confirmed that a suitable effect had been reached at this EMS concentration for anthocyanin variation (Figure 2B,D). In the current study, more than 50 putative mutant lines were classified into two groups with opposite phenotypes: *white* mutant and *red* mutant. *White* mutants were visibly confirmed with pale or no anthocyanin production at epidermis of their storage roots under sunlight; *red* mutants constitutively accumulated anthocyanin in epidermis of both aboveground and underground parts of their roots. However, high proportions of these mutants were segregated and recovered to wild type in M_3_ generation, that may be due to dominant mutations or affected by environment in the M_2_ generation [37]. Therefore, along with the results of M_3_ generation, 14 stabled mutants were chosen for further study.

We performed TILLING analysis of anthocyanin related genes (Figure 5). Excepted for a nonsense mutation at N terminal of *BrMYB4* in *r30* mutant [40], we failed in identifying additional anthocyanin biosynthesis genes with another functional mutation in the rest of mutants. These results suggest that these mutants are caused by unknown genes, for which TILLING is unavailable or by known genes with unrevealed function in anthocyanin biosynthesis.

Higher level expression of anthocyanin biosynthetic genes in *r15* at underground parts and aboveground parts was observed (Figure 7), which paralleled the upward trend in anthocyanin accumulation. Interestingly, we noticed that anthocyanin-related genes at aboveground parts in *r15* were also evoked and accumulated more anthocyanin than that of at underground parts, which showed a light-regulated manner similar to WT. In *Arabidopsis*, the E3 ubiquitin ligase COP1/SPA inhibited photomorphogenesis and anthocyanin biosynthesis in darkness by degradation of HY5 and PAP proteins [46]. The capability of producing anthocyanins at underground parts of epidermis of storage roots in *r15* probably due to stabilization of HY5 and PAP proteins, displaying a similar phenotype as *cop1/spa* mutant in *Arabidopsis*. Therefore, we hypothesized that the mutated gene in *r15* probably involved in formation of ubiquitin ligase complex and partially impaired the activity of complex to increase PAP protein levels at the epidermis of underground parts of storage roots. Under sunlight, the induction of *BrHY5* further activated *BrPAP1* expression to produce more anthocyanins in *r15*. The differences on protein levels at underground parts and aboveground parts of epidermis of storage roots in *r15* would be confirmed in further investigation. In *w9* and *w68*, transcript levels of EBGs (*BrCHS, BrCHI*) and LBGs (*BrDFR*, *BrANS1* and *BrANS2*) exhibited greatly reduced, which was consistent with anthocyanin deficiency phenotype. However, the expression level of their regulator *BrMYB12* increased remarkably and *BrPAP1* expression in *w68* was enhanced five times compared to WT at the epidermis of aboveground parts of storage roots (Figure 8). The R2R3-MYB genes (for instance, *MYB12* and *MYB75/PAP1*) play important roles in anthocyanin biosynthetic pathway (Figure 5). Recent data have indicated that expression levels of these MYB genes are regulated in a HY5-dependent manner under light conditions [29,30]. We noticed that *BrHY5* exhibited a higher expression level in *w9* and *w68* mutants than WT, which probably contributes to enrichment of *BrMYB12* and *BrPAP1* expression. However, unexpected expression pattern of *BrHY5* and *BrCOP1* in these *white* mutants remained unknown. We predict the presence of other unknown genes that cooperate with BrHY5/BrCOP1 module to regulate anthocyanin accumulation in turnip, which need to be investigated in further studies. It seems that the causal gene of *w9* is likely to function at upstream of *BrCHS* and *BrCHI* to regulate coloration in swollen roots. In turnip, *BrTT8*, along with *BrPAP1* and *BrTTG1*, plays a critical role in anthocyanin accumulation via regulating expression of LBGs (*BrDFR*, *BrANS1*, *BrANS2*, and *BrUFGT*) [33]. Despite higher *BrPAP1* expression, activity of MBW complex was limited due to robustly reduced *BrTT8* expression in *w68* (Figure 8). The gene expression analysis further showed that these *white* mutants, which shared anthocyanin deficiency phenotype, are identified as distinct genetic lines.

Structural genes, which are responsible for the anthocyanin biosynthesis, are well understood in plants. However, the regulatory network of anthocyanin biosynthesis is quite complicated and regulated by many transcription factors. In this study, we chose structural genes and critical positive regulatory genes involved in anthocyanin biosynthesis pathway for analysis. However, besides these genes, negative regulatory genes such as *MYB4*, *MYBL2* and *SPL9* (Squamosa promoter binding protein-like 9) also regulate anthocyanin production in many different ways [28,47,48]. For instance, miR156 targets the *SPL9* to stabilize MBW complex for anthocyanin accumulation [48,49]. The roles of microRNAs and protein levels of anthocyanin synthesis genes in these mutants need to be confirmed in future studies.

Recently, with the development of next-generation sequencing platforms, MBS technology (Mapping-by-sequencing) becomes a powerful tool for rapid identification in EMS-induced point mutations in plants. NGS platforms are now commercially available at affordable prices, and the MBS strategy has been widely used in model species such as *Solanum lycopersicum*, *Oryza sativa* and *Arabidopsis* [50,51,52,53]. It is sufficient for rapid simultaneously map and identify causal mutations in the genomes through sequencing genomic DNA of a pool of plants selected from an appropriate segregating population [50]. Therefore, this method is cheaper and powerful in identifying mutations in our mutants. Future work would be conducted to find out mutations, study genetic reasons for anthocyanin variations and investigate gene functions in light induced anthocyanin accumulation pathway.

In this study, we isolated two types of light-independent anthocyanin mutants and confirmed that four of them (*w9*, *w68*, *w204* and *r15*) were controlled by different single recessive genes. These mutants provide resources for investigating the regulation of light-induced anthocyanin production in detail.

## 4. Materials and Methods

### 4.1. Plant Materials

*Brassica rapa* “Tsuda” are purple turnips provided from Flower Bioengineering Institute at Northeast Forestry University, Haerbin, China. Seeds were sown in August and grown in the greenhouse during winter. About 2 months after sowing, the roots of turnip initiate swelling, of which peels could be used for anthocyanin measurement and phenotypic investigation. After flowering the collected plants were hand-pollinated in the greenhouse to produce selfed progeny or crossed progeny on purpose.

### 4.2. EMS Mutagenesis and Plant Growth Conditions

Seeds were soaked in sterile water for 6 h and dried by filter paper. Then seeds were treated with different concentration of EMS range from 0.25% to 5% for screening and determining optimal concentration to construct mutant population. Seeds treated with phosphate buffer (pH 7.0) were used as control. After 16 h mutagenesis, seeds were washed by sterile water for 4 h. Then, treated seeds were sown and grown in 18 cm pots filled with soil in a greenhouse, in which the temperature was maintained around 22 °C. Germination rate (GR) were identified as the rate from number of germinated seeds compared with total number of treated seeds in each groups, and relative germination rates were the rates of GR in each experimental groups compared with that in control.

### 4.3. M_1_ and M_2_ Population Generation

EMS-treated seeds with optimal concentration of mutagen were germinated and planted in the open field. Each of the surviving M_1_ plants was bagged for selfing and individually harvested. At least ten seeds from each M_1_ line were planted in pots in the greenhouse for M_2_ phenotype observation. The phenotypes of anthocyanin accumulation in the epidermis of swollen storage roots were recorded. Then mutants in M_2_ plants were selfed for homozygous identification and screened for further research.

### 4.4. Anthocyanin Measurement

The surface part approximately 1–2 mm thick of the swollen roots in WT and mutants were peeled by punchers and weighed using scales. Three pieces of roots epidermis on each turnip were grinded to a fine powder in liquid nitrogen and soaked in 1 mL of methanol containing 1% HCl for 24 h at 4 °C. Before measurement, samples were centrifuged at 12,000 rpm at 4 °C for 15 min. Collected 0.5 mL supernatant into a new tube and diluted samples with same volume of measured buffer. Anthocyanin content was expressed as optical density (OD_530_) per gram of peels of the swollen roots. Three biological replications were used to calculate anthocyanin content in mutants and WT.

### 4.5. Genetic Analysis on Light-Independent Anthocyanin Mutants

The mutant lines were backcrossed to WT to generate F_1_ progeny, and the F_2_ progeny were derived from self-pollination of the F_1_. At least 10 individuals of F_1_ and 200 F_2_ plants were sown for genetic analysis. Anthocyanin content of each F_1_ and F_2_ plant was recorded for analysis.

### 4.6. TILLING Analysis

*Cel*I was extracted from celery to recognize and cut mismatched bases in double-stranded DNA specifically. Mutations in anthocyanin-related genes were amplified by PCR using specific primers from WT and mutant genomes. The amplified products derived from WT, mutants and the mixture of these two samples were then subjected to a complete denaturation-slow annealing program to produce heteroduplexes: 99 °C for 10 min, 70 cycles of 70–64 °C for 20 s (−0.3 °C per cycle), then held at 15 °C. The annealed PCR products were digested with *Cel*I nuclease at 45 °C for 30 min, in a 10 μL reaction mixture containing 8 μL PCR product, 0.5 μL *Cel*I, 10 mM HEPES pH 7.5, 10 mM KCl, 10 mM MgSO_4_, 0.002% Triton X-100 and 0.2 μg/mL BSA. The reaction was stopped by adding 2 μL 0.5 M EDTA (pH 8.0) to the reaction. *Cel*I-digested PCR products were separated using 2% agarose gel electrophoresis.

### 4.7. RNA Extraction and qRT-PCR

The epidermis of swollen roots of 2-month old WT and mutants were collected then grinded to a fine powder by liquid nitrogen. Total RNA was isolated with TRIzol-Reagent. Poly (dT) cDNA was reverse-transcribed and removed gDNA from 1 μg of the total RNA by TransScript One-Step gDNA Removal and cDNA Synthesis SuperMix kit (TransGen Biotech, Beijing, China). Diluted cDNA was used as the template DNA for the quantitative RT-PCR of anthocyanin biosynthetic genes (Table S4). The PCR amplification conditions were described previously [40]. The reaction was performed on ABI7500 real-time PCR system with the Power SYBR Green PCR Master Mix (Applied Biosystems, Foster City, CA, USA), according to the manufacturer’s instructions. Melting curve analysis was performed to control the purity and specificity of amplification. The transcript levels were determined by relative quantification using the turnip *BrACTIN* (AF111812.1) as the internal control and calculated using the 2^−ΔΔ*C*t^ comparative threshold cycle (*C*t) method. At least three biological and three technological replicates were used for analysis.

## Figures and Tables

**Table 1:** The germination percentage of turnip seeds with increasing concentrations of EMS treatment.

EMS Treatment (*W*/*V*)	0.00%	0.25%	0.50%	1.00%	2.00%	5.00%
Total No. of seeds	200	200	200	200	200	200
No. of germinated seeds	191	148	97	29	0	0
Germination rate (GR)	95.5%	74%	48.5%	14.5%	0%	0%
Relative germination rate	100%	77.5%	51%	15.5%	0%	0%

Germination rate (GR) was identified as the rate from number of germinated seeds compared with total number of treated seeds in each group, and relative germination rates were the rates of GR in each experimental group compared with that in control group.

**Table 2:** Segregations of F_2_ populations crossed by the WT.

Content	*r15*	*w9*	*w68*	*w204*
No. of WT plants	141	152	114	133
No. of mutant plants	55	38	45	35
Total plants	196	190	159	168
*p* value (χ^2^ = 3:1)	0.322	0.111	0.336	0.212

## Supplementary Materials

Supplementary materials can be found at www.mdpi.com/1422-0067/18/7/1288/s1.

## Abbreviations

EMS	Ethyl methane Sulfonate
CHS	Chalcone synthase
CHI	Chalcone isomerase
F3H	Flavanone 3-hydroxylase
DFR	Dihydroflavonol reductase
ANS	Anthocyanidin synthase
UFGT	UDP-flavonoid glucosyl transferase
PAP1	Production of anthocyanin pigment 1
TT8	Transparent testa 8
TTG1	Transparent testa glabra 1
HY5COP1	Elongated hypocotyl 5Constitutively photomorphogenic 1

## References

[1] Hu D.G., Sun C.H., Ma Q.J., You C.X., Cheng L., Hao Y.J. (2016). MdMYB1 regulates anthocyanin and malate accumulation by directly facilitating their transport into vacuoles in apples. Plant Physiol..

[2] Xie X.B., Li S., Zhang R.F., Zhao J., Chen Y.C., Zhao Q., Yao Y.X., You C.X., Zhang X.S., Hao Y.J. (2012). The bHLH transcription factor MdbHLH3 promotes anthocyanin accumulation and fruit colouration in response to low temperature in apples. Plant Cell Environ..

[3] Faraco M., Spelt C., Bliek M., Verweij W., Hoshino A., Espen L., Prinsi B., Jaarsma R., Tarhan E., de Boer A.H. (2014). Hyperacidification of vacuoles by the combined action of two different P-ATPases in the tonoplast determines flower color. Cell Rep..

[4] Kovinich N., Kayanja G., Chanoca A., Otegui M.S., Grotewold E. (2015). Abiotic stresses induce different localizations of anthocyanins in *Arabidopsis*. Plant Signal Behav..

[5] Hung K.T., Cheng D.G., Hsu Y.T., Kao C.H. (2008). Abscisic acid-induced hydrogen peroxide is required for anthocyanin accumulation in leaves of rice seedlings. J. Plant Physiol..

[6] Vanderauwera S., Zimmermann P., Rombauts S., Vandenabeele S., Langebartels C., Gruissem W., Inze D., van Breusegem F. (2005). Genome-wide analysis of hydrogen peroxide-regulated gene expression in *Arabidopsis* reveals a high light-induced transcriptional cluster involved in anthocyanin biosynthesis. Plant Physiol..

[7] Hu J., Chen G., Zhang Y., Cui B., Yin W., Yu X., Zhu Z., Hu Z. (2015). Anthocyanin composition and expression analysis of anthocyanin biosynthetic genes in kidney bean pod. Plant Physiol. Biochem..

[8] Yang Y., Yao G., Yue W., Zhang S., Wu J. (2015). Transcriptome profiling reveals differential gene expression in proanthocyanidin biosynthesis associated with red/green skin color mutant of pear (*Pyrus communis* L.). Front. Plant Sci..

[9] Albert N.W., Davies K.M., Lewis D.H., Zhang H., Montefiori M., Brendolise C., Boase M.R., Ngo H., Jameson P.E., Schwinn K.E. (2014). A conserved network of transcriptional activators and repressors regulates anthocyanin pigmentation in eudicots. Plant Cell.

[10] Pelletier M.K., Shirley B.W. (1996). Analysis of flavanone 3-hydroxylase in *Arabidopsis* seedlings. Coordinate regulation with chalcone synthase and chalcone isomerase. Plant Physiol..

[11] Shirley B.W., Kubasek W.L., Storz G., Bruggemann E., Koornneef M., Ausubel F.M., Goodman H.M. (1995). Analysis of *Arabidopsis* mutants deficient in flavonoid biosynthesis. Plant J..

[12] Tohge T., Nishiyama Y., Hirai M.Y., Yano M., Nakajima J., Awazuhara M., Inoue E., Takahashi H., Goodenowe D.B., Kitayama M. (2005). Functional genomics by integrated analysis of metabolome and transcriptome of *Arabidopsis* plants over-expressing an MYB transcription factor. Plant J..

[13] Carey C.C., Strahle J.T., Selinger D.A., Chandler V.L. (2004). Mutations in the pale aleurone color1 regulatory gene of the Zea mays anthocyanin pathway have distinct phenotypes relative to the functionally similar transparent testa GLABRA1 gene in *Arabidopsis thaliana*. Plant Cell.

[14] Zhou B., Wang Y., Zhan Y., Li Y., Kawabata S. (2013). Chalcone synthase family genes have redundant roles in anthocyanin biosynthesis and in response to blue/UV-A light in turnip (*Brassica rapa; Brassicaceae*). Am. J. Bot..

[15] Petroni K., Tonelli C. (2011). Recent advances on the regulation of anthocyanin synthesis in reproductive organs. Plant Sci..

[16] Xu W., Grain D., Bobet S., Le Gourrierec J., Thevenin J., Kelemen Z., Lepiniec L., Dubos C. (2014). Complexity and robustness of the flavonoid transcriptional regulatory network revealed by comprehensive analyses of MYB-bHLH-WDR complexes and their targets in *Arabidopsis* seed. New Phytol..

[17] Stracke R., Jahns O., Keck M., Tohge T., Niehaus K., Fernie A.R., Weisshaar B. (2010). Analysis of production of flavonol glycosides-dependent flavonol glycoside accumulation in *Arabidopsis thaliana* plants reveals MYB11-, MYB12- and MYB111-independent flavonol glycoside accumulation. New Phytol..

[18] Gonzalez A., Zhao M., Leavitt J.M., Lloyd A.M. (2008). Regulation of the anthocyanin biosynthetic pathway by the TTG1/bHLH/MYB transcriptional complex in *Arabidopsis* seedlings. Plant J..

[19] Li S. (2014). Transcriptional control of flavonoid biosynthesis. Plant Signal. Behav..

[20] Zhang H.N., Li W.C., Wang H.C., Shi S.Y., Shu B., Liu L.Q., Wei Y.Z., Xie J.H. (2016). Transcriptome profiling of light-regulated anthocyanin biosynthesis in the pericarp of litchi. Front. Plant Sci..

[21] Bai S., Sun Y., Qian M., Yang F., Ni J., Tao R., Li L., Shu Q., Zhang D., Teng Y. (2017). Transcriptome analysis of bagging-treated red chinese sand pear peels reveals light-responsive pathway functions in anthocyanin accumulation. Sci. Rep..

[22] Jiang M., Ren L., Lian H., Liu Y., Chen H. (2016). Novel insight into the mechanism underlying light-controlled anthocyanin accumulation in eggplant (*Solanum melongena* L.). Plant Sci..

[23] Ahmad M., Cashmore A.R. (1997). The blue-light receptor cryptochrome 1 shows functional dependence on phytochrome A or phytochrome B in *Arabidopsis thaliana*. Plant J..

[24] Meng L.-S., Liu A. (2015). Light signaling induces anthocyanin biosynthesis via AN3 mediated COP1 expression. Plant Signal Behav..

[25] Wade H.K., Bibikova T.N., Valentine W.J., Jenkins G.I. (2001). Interactions within a network of phytochrome, cryptochrome and UV-B phototransduction pathways regulate chalcone synthase gene expression in *Arabidopsis* leaf tissue. Plant J..

[26] Chatterjee M., Sharma P., Khurana J.P. (2006). Cryptochrome 1 from brassica napus is up-regulated by blue light and controls hypocotyl/stem growth and anthocyanin accumulation. Plant Physiol..

[27] Su N., Lu Y., Wu Q., Liu Y., Xia Y., Xia K., Cui J. (2016). UV-B-induced anthocyanin accumulation in hypocotyls of radish sprouts continues in the dark after irradiation. J. Sci. Food Agric..

[28] Wang Y., Wang Y., Song Z., Zhang H. (2016). Repression of MYBL2 by both microRNA858a and HY5 leads to the activation of anthocyanin biosynthetic pathway in *Arabidopsis*. Mol. Plant.

[29] Stracke R., Favory J.J., Gruber H., Bartelniewoehner L., Bartels S., Binkert M., Funk M., Weisshaar B., Ulm R. (2010). The *Arabidopsis* bZIP transcription factor HY5 regulates expression of the PFG1/MYB12 gene in response to light and ultraviolet-B radiation. Plant Cell Environ..

[30] Shin D.H., Choi M., Kim K., Bang G., Cho M., Choi S.B., Choi G., Park Y.I. (2013). HY5 regulates anthocyanin biosynthesis by inducing the transcriptional activation of the MYB75/PAP1 transcription factor in *Arabidopsis*. FEBS Lett..

[31] Bulgakov V.P., Avramenko T.V., Tsitsiashvili G.S. (2016). Critical analysis of protein signaling networks involved in the regulation of plant secondary metabolism: Focus on anthocyanins. Crit. Rev. Biotechnol..

[32] Zhou B., Li Y., Xu Z., Yan H., Homma S., Kawabata S. (2007). Ultraviolet A-specific induction of anthocyanin biosynthesis in the swollen hypocotyls of turnip (*Brassica rapa*). J. Exp. Bot..

[33] Wang Y., Zhou B., Sun M., Li Y., Kawabata S. (2012). UV-a light induces anthocyanin biosynthesis in a manner distinct from synergistic blue + UV-B light and UV-A /blue light responses in different parts of the hypocotyls in turnip seedlings. Plant Cell Physiol..

[34] Liu C., Wang J., Huang T., Wang F., Yuan F., Cheng X., Zhang Y., Shi S., Wu J., Liu K. (2010). A missense mutation in the vhynp motif of a della protein causes a semi-dwarf mutant phenotype in *Brassica napus*. TAG Theor. Appl. Genet..

[35] Buttner B., Abou-Elwafa S.F., Zhang W., Jung C., Muller A.E. (2010). A survey of EMS-induced biennial β vulgaris mutants reveals a novel bolting locus which is unlinked to the bolting gene B. TAG Theor. Appl. Genet..

[36] Xiao X., Lin W., Li W., Gao X., Lv L., Ma F., Liu Y. (2017). The analysis of physiological variations in M2 generation of *Solanum melongena* L. Mutagenized by ethyl methane sulfonate. Front. Plant Sci..

[37] Dhaliwal A.K., Mohan A., Sidhu G., Maqbool R., Gill K.S. (2015). An ethylmethane sulfonate mutant resource in pre-green revolution hexaploid wheat. PLoS ONE.

[38] Arisha M.H., Shah S.N., Gong Z.H., Jing H., Li C., Zhang H.X. (2015). Ethyl methane sulfonate induced mutations in M2 generation and physiological variations in M_1_ generation of peppers (*Capsicum annuum* L.). Front. Plant Sci..

[39] Arisha M.H., Liang B.K., Muhammad Shah S.N., Gong Z.H., Li D.W. (2014). Kill curve analysis and response of first generation *Capsicum annuum* L. B12 cultivar to ethyl methane sulfonate. Genet. Mol. Res..

[40] Zhang L., Wang Y., Sun M., Wang J., Kawabata S., Li Y. (2014). BrMYB4, a suppressor of genes for phenylpropanoid and anthocyanin biosynthesis, is down-regulated by UV-B but not by pigment-inducing sunlight in turnip cv. Tsuda. Plant Cell Physiol..

[41] Wang J., Wang Y., Chen B., Kawabata S., Li Y. (2016). Comparative transcriptome analysis revealed distinct gene set expression associated with anthocyanin biosynthesis in response to short-wavelength light in turnip. Acta Physiol. Plant.

[42] Stephenson P., Baker D., Girin T., Perez A., Amoah S., King G.J., Ostergaard L. (2010). A rich tilling resource for studying gene function in *Brassica rapa*. BMC Plant Biol..

[43] Li H., Zhu L., Yuan G., Heng S., Yi B., Ma C., Shen J., Tu J., Fu T., Wen J. (2016). Fine mapping and candidate gene analysis of an anthocyanin-rich gene, Bnaa.PL1, conferring purple leaves in *Brassica napus* L.. Mol. Genet. Genom..

[44] Wang N., Wang Y., Tian F., King G.J., Zhang C., Long Y., Shi L., Meng J. (2008). A functional genomics resource for brassica napus: Development of an ems mutagenized population and discovery of FAE1 point mutations by tilling. New Phytol..

[45] Singer S.D., Weselake R.J., Rahman H. (2014). Development and characterization of low α-linolenic acid *Brassica oleracea* lines bearing a novel mutation in a ‘class a’ fatty acid desaturase 3 gene. BMC Genet..

[46] Maier A., Schrader A., Kokkelink L., Falke C., Welter B., Iniesto E., Rubio V., Uhrig J.F., Hulskamp M., Hoecker U. (2013). Light and the E3 ubiquitin ligase COP1/SPA control the protein stability of the MYB transcription factors PAP1 and PAP2 involved in anthocyanin accumulation in *Arabidopsis*. Plant J..

[47] Jin H., Cominelli E., Bailey P., Parr A., Mehrtens F., Jones J., Tonelli C., Weisshaar B., Martin C. (2000). Transcriptional repression by AtMYB4 controls production of UV-protecting sunscreens in *Arabidopsis*. EMBO J..

[48] Gou J.Y., Felippes F.F., Liu C.J., Weigel D., Wang J.W. (2011). Negative regulation of anthocyanin biosynthesis in *Arabidopsis* by a miR156-targeted SPL transcription factor. Plant Cell.

[49] Zhou B., Fan P., Li Y., Yan H., Xu Q. (2016). Exploring mirnas involved in blue/UV-A light response in *Brassica rapa* reveals special regulatory mode during seedling development. BMC Plant Biol..

[50] James G.V., Patel V., Nordström K.J.V., Klasen J.R., Salomé P.A., Weigel D., Schneeberger K. (2013). User guide for mapping-by-sequencing in *Arabidopsis*. Genome Biol..

[51] Fekih R., Takagi H., Tamiru M., Abe A., Natsume S., Yaegashi H., Sharma S., Sharma S., Kanzaki H., Matsumura H. (2013). Mutmap+: Genetic mapping and mutant identification without crossing in rice. PLoS ONE.

[52] Abe A., Kosugi S., Yoshida K., Natsume S., Takagi H., Kanzaki H., Matsumura H., Yoshida K., Mitsuoka C., Tamiru M. (2012). Genome sequencing reveals agronomically important loci in rice using mutmap. Nat. Biotechnol..

[53] Garcia V., Bres C., Just D., Fernandez L., Tai F.W.J., Mauxion J.P., Paslier M.C.L., Bérard A., Brunel D., Aoki K. (2016). Rapid identification of causal mutations in tomato ems populations via mapping-by-sequencing. Nat. Protoc..

