# Supplementary material for: Identification of Light-Independent Anthocyanin Biosynthesis Mutants Induced by Ethyl Methane Sulfonate in Turnip “Tsuda” (Brassica rapa)
Source: Int J Mol Sci. 2017 Jun 22;18(7):1288. doi: 10.3390/ijms18071288 (PMC5535824; doi:10.3390/ijms18071288)
Supplement: Supplementary file 1 [file ijms-18-01288-s001.pdf]

Identification of Light-independent Anthocyanin Mutants Induced by Ethyl methane Sulfonate in Turnip 'Tsuda' (*Brassica rapa*)

Table S1. Phenotypes of F1 progeny in test cross between *white* mutants

	w1	***w3***	***w9***	***w68***	***w146***	***w204***
w1		N/A	Wild-type	Wild-type	Wild-type	Wild-type
w3			N/A	Wild-type	N/A	Wild-type
w9				Wild-type	Wild-type	Wild-type
w68					Wild-type	Wild-type
w146						N/A

Table S2. Phenotypes of F1 progeny in test cross between *red* mutants

r1	***r10***	***r15***	***r21***	***r30***	***r37***	***r53***	***r57***
r1	Wild-type	Wild-type	N/A	Wild-type	Wild-type	Wild-type	Wild-type
r10		Red	Wild-type	Wild-type	Wild-type	Wild-type	Red
r15			Wild-type	Wild-type	Wild-type	Wild-type	Red
r21				Wild-type	Wild-type	N/A	Wild-type
r30					Wild-type	N/A	Wild-type
r37						Wild-type	Wild-type
r53							Wild-type

Ten F1 seeds of cross combinations were sown for phenotype observation. Some combinations without identification were failed to get F1 seeds.

Table S3. Anthocyanin biosynthetic genes (ABGs) identified in *B. rapa*

**A.** ***thaliana***	***B.rapa***	**A.** ***thaliana***	***B.rapa***
	BrACTIN(AF111812.1)	*Regulatory genes*	
Structural genes		*AtMYB12* (AT2G47460)	*BrMYB12* (Bra004456)
AtCHS (AT5G13930)	*BrCHS1* (Bra006224)<br>*BrCHS4* (Bra008792)<br>*BrCHS5* (Bra023441)	*AtPAP1* (AT1G56650)	*BrPAP1* (Bra004162)
AtCHI (AT3G55120)	*BrCHI* (Bra007142)	*AtTT8* (AT4G09820)	*BrTT8* (Bra037887)
AtF3H (AT3G51240)	*BrF3H* (Bra036828)	*AtTTG1* (AT5G24520)	*BrTTG1* (Bra009770)
AtDFR (AT5G42800)	*BrDFR* (Bra027457)	*Light responsive gene*	
AtANS (AT4G22880)	*BrANS1* (Bra013652)<br>*BrANS2* (Bra019350)	*AtHY5* (AT5G11260)	*BrHY5* (Bra008976)
AtUGT79B1 (AT5G54060)	*BrUFGT* (Bra003021)	*AtCOP1*(AT2G32950)	*BrCOP1*(Bra005541)

Table S4. The DNA sequences of PCR primers used for real-time PCR of anthocyanin biosynthetic and regulatory genes

Target gene	Forward primers	Reverse primers
BrACTIN	GCTCAGTCCAAGAGAGGTATTC	GCTCGTTGTAGAAAGTGTGATG
BrCHS	GGGACTCACCTTCCATCTCCTC	CGCGTGGCTCTCATCTTCTCT
BrCHI	TTCACCGTCCACCATCGTCT	GACATAATACAACAACATAAAACACACC
BrF3H	CGAAAGAAGAACATGACCACAAG	CACAACCGAACCAACACAATAG
BrF3'H	GTTTAGGGTTACGGACGATTCA	TCTCTTCCATGTTCAGCTTCTC
BrDFR	TGGTGCCAAGGGACGTTATG	TTGCCTGAGAAACTCGGAGATAG
BrANS1	CCTCCAAGGACGTTTGCTCA	GACTTCATCCTTTTTCTCAGTTACC
BrANS2	GCTGAGACTCCGGCTAAGTTTCC	TTTTGTCTCAGGCACCAACTCC
BrUFGT	CGGAGGTGTACCGATGATTTG	ACAGCCTCCACCGCTCTTC
BrPAP1	AACTTTGGAGCCTGTTGGATG	TCACAATGTCACGCACAAGCA
BrTT8	CGACAATCATTTTGAGGCAGAG	TCAGCAATGGTTGGTTTCTTCC
BrTTG1	CTCTTATCTGGGAGTTGCCGAC	GCAATGCCAATCCAATCAGG
BrMYB4	GGCAAAGAAAGAGACCAACA	AAAAGTTTGTACAGTTACGC
BrMYB12	CGGGCAAAGTAACTGCGAAA	GACAGAAGCCAAGCGACCAAC
BrHY5	CAAGTTTTGGGTTGTTTTTGGGTG	AAAGAGGTCAGAGGCTCGCATC
BrCOP1	AAAAGCGGCGTCAGTTGGT	CCGTGGCTGTATCCTTCCCTA
